# Long-Term Outcomes in Percutaneous Radiofrequency Ablation for Histologically Proven Colorectal Lung Metastasis

**DOI:** 10.1007/s00270-020-02623-1

**Published:** 2020-08-18

**Authors:** Jim Zhong, Ebrahim Palkhi, Helen Ng, Kevin Wang, Richard Milton, Nilanjan Chaudhuri, James Lenton, Jonathan Smith, Bobby Bhartia, Tze Min Wah

**Affiliations:** 1 Department of Diagnostic and Interventional Radiology, Institute of Oncology, St. James’s University Hospital, Leeds Teaching Hospitals NHS Trust, Leeds, LS9 7TF UK; 2 University of Leeds, Woodhouse, Leeds, LS2 9JT UK; 3 Department of Thoracic Surgery, Institute of Oncology, St. James’s University Hospital, Leeds Teaching Hospitals NHS Trust, Leeds, LS9 7TF UK

**Keywords:** Radiofrequency ablation, Colorectal, Metastases, Lung, Overall survival, Progression-free survival

## Abstract

**Introduction:**

To evaluate the long-term outcome of image-guided radiofrequency ablation (RFA) when treating histologically confirmed colorectal lung metastasis in terms of overall survival (OS), progression-free survival (PFS) and local tumour control (LTC).

**Materials and Methods:**

Retrospective single-centre study. Consecutive RFA treatments of histologically proven lung colorectal metastases between 01/01/2008 and 31/12/14. The primary outcome was patient survival (OS and PFS). Secondary outcomes were local tumour progression (LTP) and complications. Prognostic factors associated with OS/ PFS were determined by univariate and multivariate analyses.

**Results:**

Sixty patients (39 males: 21 females; median age 69 years) and 125 colorectal lung metastases were treated. Eighty percent (*n* = 48) also underwent lung surgery for lung metastases. Mean metastasis size (cm) was 1.4 ± 0.6 (range 0.3–4.0). Median number of RFA sessions was 1 (1–4). During follow-up (median 45.5 months), 45 patients died (75%). The estimated OS and PFS survival rates at 1, 3, 5, 7, 9 years were 96.7%, 74.7%, 44.1%, 27.5%, 16.3% (median OS, 52 months) and 66.7%, 31.2%, 25.9%, 21.2% and 5.9% (median PFS, 19 months). The LTC rate was 90% with 6 patients developing LTP with 1-, 2-, 3- and 4-year LTP rates of 3.3%, 8.3%, 10.0% and 10.0%. Progression-free interval < 1 year (*P* = 0.002, HR = 0.375) and total number of pulmonary metastases (≥ 3) treated (*P* = 0.037, HR = 0.480) were independent negative prognostic factors. Thirty-day mortality rate was 0% with no intra-procedural deaths.

**Conclusion:**

The long-term OS and PFS following RFA for the treatment of histologically confirmed colorectal lung metastases demonstrate comparable oncological durability to surgery.

**Electronic supplementary material:**

The online version of this article (10.1007/s00270-020-02623-1) contains supplementary material, which is available to authorized users.

## Introduction

Colorectal cancer is the second most common cause of cancer death worldwide [1]. A quarter of colorectal patients who undergo curative resection will develop distant metastases with the lung being the second most common site [2]. A large epidemiological study of colorectal cancer patients with lung metastases found 3- and 5-year survival rates of 1.3% and 6.9%, highlighting the poor prognosis in this cohort of patients if left untreated [3]. When the metastatic disease is localised to two or less visceral sites (oligometastatic disease), a potentially curative approach exists. Localised resection of lung metastases is the widely recognised standard of care for patients with oligometastatic disease from colorectal cancer despite the lack of randomised control trial (RCT) data comparing treatment options [4].

Percutaneous image-guided thermal ablation with radiofrequency ablation (RFA) has been widely used in the last decade and is established as a potential alternative to surgical resection [5, 6]. It is the most suitable for patients with small (< 2 cm) lung metastases and in those who may not be a surgical candidate due to their comorbidities [5, 7]. RFA of lung metastases offers a minimally invasive, repeatable treatment, with better preservation of lung function treatment when compared to surgical resection [8]. No studies directly compare RFA with surgery, but published results suggest local control and survival rates similar to surgery [8, 9]. Long-term survival data on RFA remain limited when compared to the surgical literature, making it difficult to draw definitive conclusions on the long-term oncological durability of the treatment [10].

The aim of this study was to report the long-term survival outcomes of image-guided RFA when treating histologically confirmed colorectal lung metastasis in terms of overall survival (OS), progression-free survival (PFS) and local tumour control (LTC).

## Methods

### Study Population

The study involved retrospective analysis of a prospectively collected database. It was performed under a waiver of informed consent and ethics approval by the institutional review board.

All consecutive image-guided pulmonary RFA procedures undertaken between 1 January 2008 and 31 December 2014 were reviewed. Inclusion criteria were treatment for oligometastatic colorectal metastases with histological confirmation either from surgical resection or percutaneous image-guided biopsy at the time of or prior to ablation of at least one lung lesion. Patients without histological confirmation and those with other primary pathologies were excluded from this study.

All treatments were performed in a single regional tertiary cancer centre. The lung metastases treatment decisions were made by the local multidisciplinary team (MDT) which included thoracic surgery and interventional oncology. Treatment decisions were made on the basis of the likelihood of technical success, preservation of lung function, the requirement to obtain histological confirmation of disease, the performance status of the patient and the patient’s choice. At the time of the study, stereotactic radiotherapy (SABR) had not been commissioned for the treatment of lung metastases.

### RFA Procedure and Follow-up

All treatments were targeted with computed tomography (CT) guidance and performed under general anaesthesia (GA). The RFA device utilised a unipolar multi-tined expandable applicator that delivered alternating RF current controlled by the impedance in the RF treatment algorithm (LeVeen; Boston Scientific, Natick, MA).

The aim of treatment was to ablate the lung metastasis with a minimum of a 5-mm treatment margin. Lesions in the same lung were treated in a single session, but contralateral lesions were treated at a different treatment session due to the risk of pneumothorax. An immediate post-RFA unenhanced chest CT was obtained after RFA electrode removal to assess for pneumothorax. All pneumothoraces were observed for up to 10 min under GA, and enlargement was considered an indication for drainage. All patients were observed overnight following RFA for potential complications, and a chest radiograph was performed 4 h post-RFA and before discharge to ensure there was no delayed or worsening pneumothorax.

An unenhanced chest CT scan was performed at 1, 3, 6, 12, 18, 24 months and then yearly after the RFA to evaluate the local control rate, the presence of new metastatic disease within the lungs and for any complications. All patients with local tumour progression were reassessed by consensus of the local MDT for suitability of surgical resection or repeat image-guided thermal ablation eligibility.

### Definitions of Study Outcome Measures

Standardised definitions of outcomes and grading of complications were applied [5, 11, 12].

Technical success of RFA was defined as the complete coverage of the tumour by the ablation zone of ground glass opacity (GGO), with at least a 5-mm ablative treatment margin seen on the CT imaging at the end of the procedure. GGO was defined as increased opacification of lung, with preservation of bronchial and vascular margins as per the Fleischner Society [13]. Technical failure was defined as incomplete tumour ablation with the presence of residual tumour at the end of the RFA treatment session not encompassed within the ablation zone or with less than 5-mm ablative treatment margin.

Local tumour progression (LTP) was defined by the appearance of tumour foci inside or at the edge of the ablation zone during imaging follow-up, provided that complete ablation with adequate margins could be documented with a previous study.

Time to progression (TTP) is the time interval between first RFA and local or distant disease progression. Its associated metric is progression-free survival (PFS) defined as the interval from RFA to local or distant disease progression or death (from any cause); both are measures of oncologic efficacy rather than technical success. Overall survival (OS) was defined as the time from first treatment with RFA to death (from any cause). RFA-related adverse events were assessed according to the CIRSE classification system [12].

### Statistical Analysis

Statistical analyses were performed using SPSS software (version 23.0; IBM Corp, Armonk, New York). Kaplan–Meier method used to evaluate OS and PFS with 95% confidence intervals (CI) was calculated. To detect prognostic factors the following variables were collected: sex (male or female), progression-free interval (< 1 year or ≥ 1 year), history of lung surgery for metastases (yes or no), total number of pulmonary metastases treated by RFA and surgery (< 3 or ≥ 3), largest size of ablated tumour (< 2 cm or ≥ 2 cm), history of liver metastases (yes or no) and chemotherapy prior to RFA (yes or no) (Supplementary material). Univariate analyses were performed by the log-rank test to compare the survival rates (for both OS and PFS) between each pair of groups/prognostic factors (Supplementary material). Multivariate analyses to determine the independent prognostic factors were performed using the Cox proportional-hazards model. For all analyses, a *P* value of  < 0.05 was considered statistically significant.

## Results

A total of 96 patients underwent RFA between 1 January 2008 and 31 December 2014. In total, 60 patients with histologically confirmed colorectal lung metastases (*n* = 125) treated by image-guided RFA were included in the retrospective analysis. Patient characteristics are displayed in Table 1. The 36 patients excluded were due to lack of histological confirmation of colorectal metastases or treatment for other disease pathologies. The mean metastasis size was 1.4 cm. Standard deviation (SD) was 0.6 cm and ranges 0.3–4.0 cm.

**Table 1:** Patient characteristics

Characteristic	Value (%/ range)
Age (y)	
Median	69 (31–89)
Sex	
Male	39 (65%)
Female	21 (35%)
Site of primary cancer	
Colon	28 (46.7%)
Sigmoid	5 (8.3%)
Rectum	27 (45%)
History of lung surgery for metastases	
Yes	48 (80%)
No	12 (20%)
Chemotherapy prior to RFA	
Yes	21 (35%)
No	39 (65%)
History of colorectal liver metastases	
Yes	28 (47%)
No	32 (53%)
Number of lesions treated with RFA	Total = 125
Median	2 (1–9)
Number of lesions treated with surgery	Total = 143
Median	2 (1–10)
Number of RFA sessions	Total = 82
Median	1 (1–4)
Maximum size of ablated tumour	
0–1 cm	71 (57%)
1–2 cm	48 (38%)
2–3 cm	5 (4%)
3–4 cm	1 (1%)

The median duration of follow-up after the initial image-guided RFA was 45.5 months (range 3–141).

Out of the 28 patients who had a history of colorectal liver metastases (Table 1), 27 had locoregional liver treatment with either liver ablation or resection for their colorectal metastases.

The maximum number of lung metastases treated with image-guided RFA was 9, and this patient had two separate treatment sessions to treat left lower lobe nodules (*n* = 5) followed by a further treatment session to treat the left upper lobe nodules (*n* = 4).

### Local Tumour Progression and Long-Term Survival Outcomes

Out of 125 treated pulmonary metastases, the primary technical success rate in this cohort was 99.2%. One patient had incomplete treatment of a metastasis (technical failure) and required a second RFA to successfully treat the residual disease. The overall technical success rate for this cohort of patients treated with image-guided RFA for their colorectal lung metastasis was 100%

The overall LTC rate was 90% with 10% of the patients (*n* = 6) developing LTP with a median time to local progression of 18 months (range 10–26) from the initial treatment. The LTP rate was 3.3% at 1 year, 8.3% at 2 years, 10.0% at 3 years and 10.0% at 4 years.

During the study review period (01/01/2008–31/12/2019), a total of 45 patients with colorectal lung metastasis treated with image-guided RFA (75%) died. The causes were disease progression (*n* = 29), heart failure (*n* = 2), decompensated liver disease (*n* = 1) or unknown causes (*n* = 13).

**Fig. 1:**
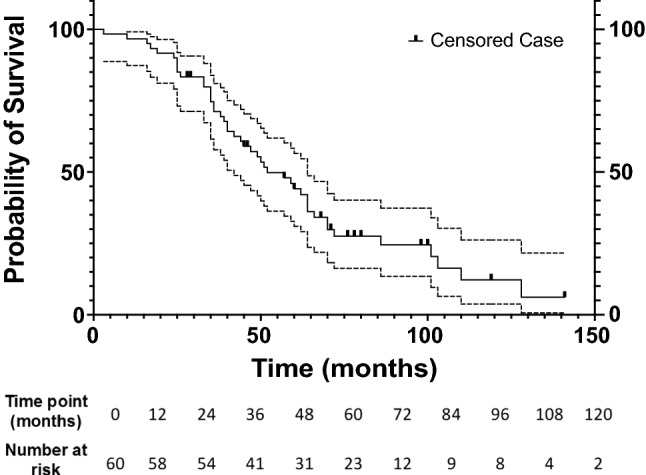
Graph shows Kaplan–Meier overall survival estimate for all patients with metastasis to lung from colorectal carcinoma treated with RFA. Dotted lines represent 95% confidence intervals

The Kaplan–Meier survival analysis for all patients (*n* = 60) found the median OS to be 52 months (95% CI 39.3–64.7) (Fig. 1). The 1-, 3-, 5-, 7- and 9-year overall survival rates were 96.7%, 74.7%, 44.1%, 27.5 and 16.3%.

**Fig. 2:**
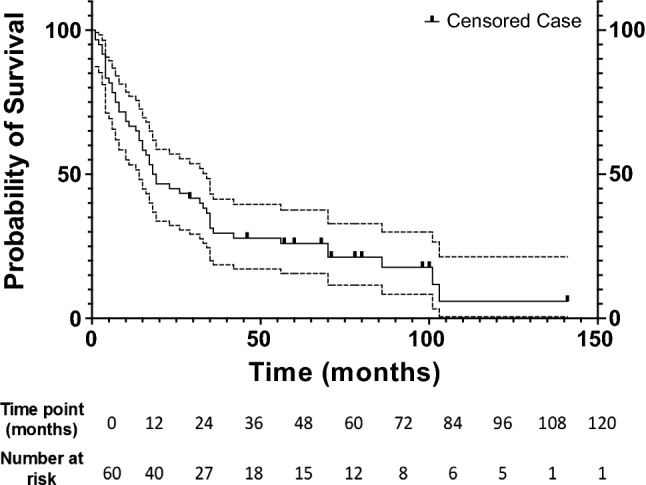
Graph shows Kaplan–Meier progression-free survival estimate for all patients with metastasis to lung from colorectal carcinoma treated with RFA. Dotted lines represent 95% confidence intervals

Median PFS was 19 months (95% CI 9.6–28.4) (Fig. 2). The 1-, 3-, 5-, 7- and 9-year progression-free survival rates were 66.7%, 31.2%, 25.9%, 21.2% and 5.9%.

Univariate and multivariate analysis (detailed breakdown provided for each prognostic factor is provided in Supplementary Information) identified progression-free interval of less than one year to be significantly associated with worse OS (*P* = 0.002, hazard ratio 0.375), and the total number of pulmonary metastases (≥ 3) treated was significantly associated with worse PFS (*P* = 0.037, hazard ratio 0.480).

### Adverse Events

For this patient cohort, the 30-day post-RFA mortality rate was 0% with no intra-procedural deaths.

In total, 36 patients (60%) developed a pneumothorax (Grade 1) following RFA, and all were diagnosed on the immediate postprocedural unenhanced chest CT. Of these patients, 18 required a CT-guided chest drain insertion to relieve the enlarging pneumothorax, while the remainder were managed conservatively with spontaneous resolution. Two (3%) patients required a chest drain for a subsequent reactive effusion (Grade 2). Two patients (3%) developed a post-RFA air leak requiring video-assisted thoracoscopic surgery (Grade 3).

## Discussion

A median overall survival of 52 months with a five-year OS and PFS of 44.1% and 25.9%, respectively, is in line with existing literature. Few previous studies have examined long-term survival following RFA in colorectal metastases (Table 2) [14–18], and none included only patients with histological confirmation as in the current study. Although desirable, histological confirmation is not always feasible prior to treatment. A relevant clinical history with concordant imaging may sufficiently support an MDT decision to proceed with treatment. Biopsy at the time of ablation risks inducing haemorrhage or pneumothorax and may jeopardise accuracy of RF probe placement. One recently suggested option to mitigate these risks is to biopsy immediately after RFA which carries a diagnostic sampling rate of 90% [19].

**Table 2:** RFA studies of colorectal lung metastases reporting 5-year survival data

Authors	Year	Number of patients	Ablation modality	Overall survival rates (%)			Mean/median survival (months)
				1 y	3 y	5 y	
Simon et al. [34]	2007	18	RFA	87	57	57	–
Yamakado et al. [45]	2009	78	RFA	84	56	35	38
Matsui et al. [15]	2015	84	RFA	95.2	65	51.6	67
Ferguson et al. [35]	2015	157	RFA	89	44	19.9	33.3
de Baère et al. [7]	2015	566*	RFA	92.4	67.7	51.5	62
Vogl et al. [22]	2016	41	RFA	76.9	50.8 (2 y)	8 (4 y)	24.2
Shi et al. [36]	2017	43*	RFA	77	42	34	–

The asterisk (*) highlights inclusion of non-colorectal lung metastases

The results of this current study support the findings of previous studies without comprehensive histological confirmation. A systematic review by Lyons et al. [20] included over 900 patients from 8 RFA studies on colorectal lung metastases and demonstrated OS rates at 5 years of 19.9% and PFS rates of 7%. A more recent study by Matsui et al. published a more favourable 5-year OS rate of 52% with a median OS of 67 months [15]. Differences in the outcomes data can reflect confounders within the different patient populations. The improved long-term survival observed in more recent studies is possibly attributable to advancement in chemotherapy for metastatic colorectal cancer in the neoadjuvant and palliative setting [21].

The LTC rate in this study was 90% and a median time to LTP of 18 months (3–26). This is consistent with other studies which quote a LTC rate of 69–87% [15, 22, 23]. The median time to LTP in the published RFA literature is variable [10]. Previous studies have quoted the median times to LTP in the range of 8.2–11.2 months; however, the ranges vary widely from 2.6 to 43.7 months [15, 23]. In the largest published RFA series by de Baère et al. [7], the 1-, 2-, 3- and 4-year local tumour progression (LTP) rates were 5.9%. 8.5%, 10.2% and 11%, respectively. Our study confirms the finding that the majority of local recurrences occur within the first 2 years following treatment.

In terms of prognostic factors, this study confirms that the total number of pulmonary metastases (≥ 3) treated with RFA and surgery is associated with worse PFS (*P* = 0.037). The number of metastases has also been found to a predictor of survival in other large retrospective RFA studies [7] and surgical series [24]. The current study confirmed a previously demonstrated lack of correlation between a history of treated liver metastases with survival [17, 23].

Surgical series (Table 3) have shown favourable long-term survival rates, and despite the absence of RCT data, resection is considered first-line treatment [4]. Surgical literature focuses on 5-year survival with estimates ranging between 38.3 and 71.3% [24–33]. The ablation literature frequently quotes 1–3-year survival, but when reported the five-year survival after thermal ablation is less favourable compared to the surgical literature and the 5-year survival rates following RFA ranges between 19.9 and 57% [7, 14, 15, 34–36]. A multi-centre surgical series [33] found 5-year disease-free and OS rates of 37.1% and 68.1% (compared to 30.4% and 44.1% in the current study), over a median follow-up of 65 months. The favourable survival rates may be partly attributable to patient selection criteria with a higher proportion (75%) of patients with a solitary metastasis and only complete resections included in the results [33, 37]. Comparing ablation with surgical outcomes fails to recognize that these are frequently not mutually exclusive groups. Eighty percent of the patients in our study also had surgical resection for colorectal lung metastases. This is higher than other published cohorts, which varies from 45 to 53% [15, 23]. This is perhaps a reflection of a collaborative MDT approach where complete treatment of the lung metastases with preservation of lung function regardless of underlying fitness is one of the stated aims. Image-guided thermal ablation of lung metastases in our cancer centre acts as an adjunct rather than competitor to the existing surgical resection cancer service in this cohort of patients.

**Table 3:** Surgical studies focusing on colorectal lung metastases with reported 5-year survival data

Authors	Year	Number of patients	Overall survival rates (%)			Mean/median survival (months)
			1 y	3 y	5 y	
Kanemitsu et al. [25]	2004	313	90.4	53.0	38.3	38.4
Yedibela et al. [26]	2006	153	–	64 (2 y)	37	39
Welter et al. [27]	2007	169	–	–	39	47.2
Onaitis et al. [28]	2009	378	–	78	56	–
Blackmon et al. [24]	2012	229	–	–	55.4	70.1
Iida et al. [29]	2013	1030	–	–	53.5	69.5
Hirosawa et al. [30]	2013	266	–	–	56.5	–
Bolukbas et al. [31]	2014	165	–	–	54	64
Sun et al. [32]	2017	154	–	–	71.3	–
Okumura et al. [33]	2017	785	–	–	68.1	–

Although microwave ablation (MWA) has theoretical advantages over RFA, such as less heat sink effect and potentially more uniform ablation zones in a shorter time [22, 38–40], the LUMIRA RCT has found no statistically significant difference between MWA and RFA in terms of survival [41]. In 2019, a meta-analysis of 53 studies showed the 5-year OS rates for RFA-treated patients (*n* = 738) were higher compared with MWA-treated patients (*n* = 469) (*P* < 0.001) and treatment with RFA was correlated with a longer median OS of 34.8 months compared to 18.7 for MWA [6]. Confounding factors may be that RFA is better known and has been used for longer, with more reproducible results, whereas MWA is a newer modality with smaller study sample sizes. Larger groups with longer follow-up periods are required before firm conclusions can be drawn.

Currently, no RCT exists that compares thoracic surgery versus image-guided ablation and will be difficult to achieve. The recent Pulmonary Metastasectomy in Colorectal Cancer (PulMiCC) trial (comparing surgery vs active monitoring) had to be stopped due to failure to recruit the required number of patients [44]. An RCT in the setting of colorectal lung metastases would also be of questionable value, given many patients receive multiple different treatment modalities throughout their course. Registries can provide evidence of the technical success and safety of ablation in the context of treating metastatic disease.

The limitations of this study were the retrospective single-centre design with a small sample size. Given that only patients with histological proven cancer were included, this may have biased the results towards better outcomes if comorbid patients were not subjected to biopsy due to the inherent small additional risks of lung biopsy.

## Conclusion

The long-term OS and PFS following RFA for the treatment of histologically confirmed colorectal lung metastases demonstrate comparable oncological durability to surgery.

## Electronic supplementary material

Below is the link to the electronic supplementary material.

Supplementary file 1 (DOCX 112 kb)
