# Supplementary material for: Long-Term Outcomes in Percutaneous Radiofrequency Ablation for Histologically Proven Colorectal Lung Metastasis
Source: Cardiovasc Intervent Radiol. 2020 Aug 18;43(12):1900–7. doi: 10.1007/s00270-020-02623-1 (PMC7649179; doi:10.1007/s00270-020-02623-1)
Supplement: Supplementary file 1 — (DOCX 112 kb) [file 270_2020_2623_MOESM1_ESM.docx]

**Electronic Supplementary Material**

Table 1: Univariate and Multivariate analyses of potential prognostic factors for OS

|  | Univariate Analysis | | | | | | | Multivariate Analysis | | |
| --- | --- | --- | --- | --- | --- | --- | --- | --- | --- | --- |
|  |  | Overall Survival (%) | | | | |  |  |  |  |
| Variable | No. of Pts. | 1 y | 3 y | 5 y | 7 y | 9 y | P Value | HR | 95% CI | P Value |
| All patients | 60 | 96.7 | 74.7 | 44.1 | 27.5 | 16.3 |  |  |  |  |
| Sex |  |  |  |  |  |  |  |  |  |  |
| Male | 39 | 95.2 | 57.1 | 42.9 | 26.6 | 16.6 | 0.973 | 0.705 | 0.336-1.478 | 0.355 |
| Female | 21 | 97.4 | 84.3 | 47.9 | 30.6 | 15.3 |  |  |  |  |
| Progression-free interval |  |  |  |  |  |  |  |  |  |  |
| < 1 y | 22 | 90.1 | 45.5 | 21.2 | 10.6 | 10.6 | 0.001 | 0.220 | 0.095-0.508 | <0.001 |
| ≥ 1 y | 38 | 100.0 | 86.2 | 57.5 | 37.3 | 19.6 |  |  |  |  |
| History of lung surgery for metastases |  |  |  |  |  |  |  |  |  |  |
| Yes | 48 | 95.8 | 81.1 | 43.1 | 27.4 | 23.5 | 0.840 | 0.921 | 0.366-2.317 | 0.861 |
| No | 12 | 100.0 | 75.0 | 47.6 | 28.6 | 0.0 |  |  |  |  |
| Total number of pulmonary metastases treated |  |  |  |  |  |  |  |  |  |  |
| < 3 | 32 | 96.9 | 71.5 | 50.6 | 33.0 | 8.8 | 0.863 | 1.603 | 0.720-3.565 | 0.248 |
| ≥ 3 | 28 | 96.4 | 78.3 | 41.0 | 21.7 | 14.5 |  |  |  |  |
| Largest size of ablated tumour |  |  |  |  |  |  |  |  |  |  |
| < 2 cm | 50 | 98.0 | 79.6 | 45.3 | 28.0 | 12.0 | 0.963 | 0.665 | 0.263-1.681 | 0.389 |
| ≥ 2 cm | 10 | 90.0 | 50.0 | 40.0 | 26.7 | - |  |  |  |  |
| History of liver metastases |  |  |  |  |  |  |  |  |  |  |
| Yes | 27 | 96.3 | 74.1 | 43.3 | 19.5 | - | 0.798 | 0.879 | 0.439-1.760 | 0.715 |
| No | 33 | 93.9 | 72.5 | 45.2 | 33.9 | 17.4 |  |  |  |  |
| Chemotherapy before RFA |  |  |  |  |  |  |  |  |  |  |
| Yes | 21 | 95.2 | 81.0 | 52.4 | 30.6 | 20.4 | 0.501 | 0.919 | 0.477-1.773 | 0.802 |
| No | 39 | 94.9 | 71.0 | 39.2 | 26.1 | 14.5 |  |  |  |  |

Table 2: Univariate and Multivariate analyses of potential prognostic factors for PFS

|  | Univariate Analysis | | | | | | | Multivariate Analysis | | |
| --- | --- | --- | --- | --- | --- | --- | --- | --- | --- | --- |
|  |  | Progression Free Survival (%) | | | | |  |  |  |  |
| Variable | No. of Pts. | 1 y | 2 y | 3 y | 4 y | 5 y | P Value | HR | 95% CI | P Value |
| All patients | 60 | 66.7 | 45.0 | 31.2 | 27.8 | 25.9 |  |  |  |  |
| Sex |  |  |  |  |  |  |  |  |  |  |
| Male | 39 | 64.1 | 43.6 | 24.7 | 22.0 | 18.8 | 0.166 | 1.636 | 0.849-3.150 | 0.141 |
| Female | 21 | 71.4 | 47.6 | 38.1 | 38.1 | 38.1 |  |  |  |  |
| History of lung surgery for metastases |  |  |  |  |  |  |  |  |  |  |
| Yes | 48 | 60.4 | 39.6 | 22.1 | 22.1 | 19.9 | 0.140 | 1.068 | 0.453-2.514 | 0.881 |
| No | 12 | 91.7 | 66.7 | 58.3 | 50.0 | 50.0 |  |  |  |  |
| Total number of pulmonary metastases treated |  |  |  |  |  |  |  |  |  |  |
| < 3 | 32 | 81.3 | 65.6 | 44.9 | 42.7 | 39.1 | 0.005 | 0.480 | 0.241-0.955 | 0.037 |
| ≥ 3 | 28 | 50.0 | 21.4 | 10.7 | 10.7 | 10.7 |  |  |  |  |
| Largest size of ablated tumour |  |  |  |  |  |  |  |  |  |  |
| < 2 cm | 50 | 72.0 | 48.0 | 29.3 | 29.3 | 27.1 | 0.344 | 1.466 | 0.651-3.302 | 0.356 |
| ≥ 2 cm | 10 | 40.0 | 30.0 | 30.0 | 30.0 | 30.0 |  |  |  |  |
| History of liver metastases |  |  |  |  |  |  |  |  |  |  |
| Yes | 27 | 66.7 | 40.7 | 18.5 | 18.5 | 14.8 | 0.353 | 1.102 | 0.586-2.073 | 0.762 |
| ≥ 2 cm | 10 | 40.0 | 30.0 | 30.0 | 30.0 | 30.0 |  |  |  |  |
| Chemotherapy before RFA |  |  |  |  |  |  |  |  |  |  |
| Yes | 21 | 61.9 | 38.1 | 28.6 | 28.6 | 28.6 | 0.476 | 1.165 | 0.583-2.327 | 0.665 |
| No | 39 | 71.2 | 50.1 | 38.7 | 38.7 | 34.8 |  |  |  |  |
